# A Computational Approach to Analyze the Mechanism of Action of the Kinase Inhibitor Bafetinib

**DOI:** 10.1371/journal.pcbi.1001001

**Published:** 2010-11-18

**Authors:** Thomas R. Burkard, Uwe Rix, Florian P. Breitwieser, Giulio Superti-Furga, Jacques Colinge

**Affiliations:** Research Center for Molecular Medicine of the Austrian Academy of Science, Vienna, Austria; University of California San Diego, United States of America

## Abstract

Prediction of drug action in human cells is a major challenge in biomedical research. Additionally, there is strong interest in finding new applications for approved drugs and identifying potential side effects. We present a computational strategy to predict mechanisms, risks and potential new domains of drug treatment on the basis of target profiles acquired through chemical proteomics. Functional protein-protein interaction networks that share one biological function are constructed and their crosstalk with the drug is scored regarding function disruption. We apply this procedure to the target profile of the second-generation BCR-ABL inhibitor bafetinib which is in development for the treatment of imatinib-resistant chronic myeloid leukemia. Beside the well known effect on apoptosis, we propose potential treatment of lung cancer and IGF1R expressing blast crisis.

## Author Summary

Protein interaction data are accumulating rapidly and, although imperfect and incomplete, they provide a valuable global description of the complex interplay of proteins in a human cell. In parallel, modern proteomics technologies make it possible to measure in an unbiased manner the protein targets of a drug. Such data reveal multiple targets in a view that contrasts with a previously prevalent paradigm that drugs had single – or a very limited number of – targets. In this context of newly available systems level data and more precise and complete information about drug interactions, it is natural to try to determine the global perturbation exerted by a drug on a human cell to identify potential side effects and additional indications. We present a computational method that aims at making such predictions and apply it to bafetinib, a recently developed leukemia drug. We show that meaningful predictions of additional applications to other cancers or resistant cases and likely side effects are obtained that are not straightforward to determine with existing algorithms. Our method has a strong potential to be applicable to other drugs.

## Introduction

**Figure 1:**
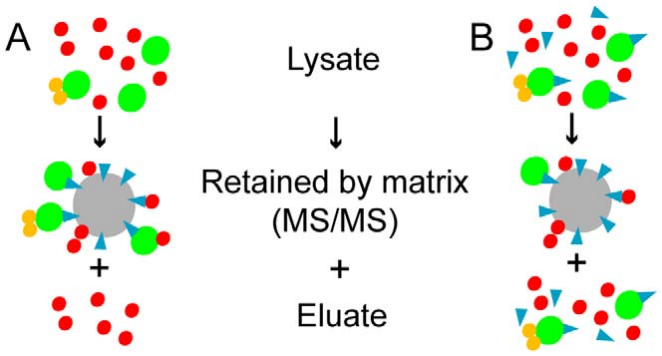
Chemical proteomics without (A) and with (B) soluble drug competition. (A) The drug (blue) bound to a matrix (grey) retains the drug targets (green) and secondary binders (orange). Most unspecific proteins (red) are washed of but some could stick to the matrix. The retained proteins are analyzed with MS/MS. (B) If the soluble drug is supplemented, it blocks the binding pocket of its target yielding a reduced amount of pulled-down proteins that are specific drug binders. Only sticky unspecific proteins and weak drug targets are retained.

Biomedical research is changing towards a systems pharmacology view of drug action [1]. In parallel, chemical proteomics (Figure 1), a postgenomic version of classical drug affinity purifications which use is growing rapidly, has been developed to measure drug target profiles in an unbiased manner [2]–[5]. It usually reveals larger than expected spectra of targets which are causing both therapeutic and adverse effects. Such unbiased target profiles are very valuable entry points to understand which regions of the cell machinery are perturbed by a drug. It is hence desirable to develop new specific algorithms exploiting chemical proteomics profiles. Generally, it is natural that protein interaction networks are involved to characterize drug targets, action on diseases, and potential side effects [6]–[11]. Existing methods are mainly based on the network topology and on an integration of gene expression data and phenotype similarities [12]–[14].

Alternatively, precise modeling of perturbations which change the protein interaction network has the potential to predict new drug targets and to provide a detailed mechanism of action simultaneously [15]–[17]. Beside network approaches, classical gene ontology (GO) enrichment analyses of drug targets are commonly used which result in no detailed mechanism but identify different processes and functions of direct involvement [11], [18]. However, one pivotal aspect is that drug targets can perturb protein interaction networks and biological processes without being directly part of the latter. Therefore, we present a new algorithm which combines direct and peripheral perturbations of functional sub-networks and exploits chemical proteomics drug target profiles. The idea of functional sub-networks is based on the finding that genes associated with the same disease often share protein-protein interactions and gene ontology terms [19]. Our algorithm estimates the drug impact on biological processes and the detailed perturbation effects can be visualized as a network, which facilitates interpretation. Furthermore, we introduce an affinity score to weigh the drug target profile on the basis of interaction strengths.

We applied our algorithm to the bafetinib (NS-187, INNO-406) target profile. Bafetinib is a small molecule tyrosine kinase inhibitor in development for chronic myeloid leukemia (CML) [20]. It has been designed to potently and specifically inhibit BCR-ABL and the SRC family kinase (SFK) LYN, but no other SFKs, with the purpose of displaying an improved safety profile over multi-kinase and pan-SFK inhibitors, such as dasatinib, while retaining the advantageous dual mechanism of action. We have recently characterized the detailed target profile of bafetinib by chemical proteomics and to interpret the complex dataset obtained is challenging. One of the most popular methods for distinguishing (and potentially quantifying) specific drug targets from non-specific background proteins is the competition of soluble drug molecules with the affinity matrix for drug binding proteins (Figure 1) [21]–[23]. Comparison of the protein eluates from a competed and a non-competed drug pulldowns will highlight specific binders, while non-specific binding proteins will not be affected. However, even after correct identification and potentially determination of quantitative interaction parameters for distinct drug-protein pairs, a global or mechanistic understanding of drug effects is but a distant goal requiring some sophisticated experimental and/or theoretical follow-up. Our theoretical effort advances significantly our mechanistic understanding of the effects of bafetinib and provides others with a computational strategy applicable to different drug profiles.

## Materials and Methods

Our computational approach to predict the impact of bafetinib on a functional network is based on the human protein-protein interaction network, on the annotation of its nodes and on a drug target profile associated with an affinity measure.

### Human protein interaction network

The network is constructed from protein-protein interactions found in the public interaction databases HPRD, MINT, Intact, DIP and BioGRID [24]–[28]. Furthermore, it is supplemented with published interactions of the BCR-ABL core complex which is the primary target of bafetinib in chronic myeloid leukemia (CML) [29]. The resulting undirected network contains 11505 proteins and 80363 interactions.

### Uniform functional sub-network

The human network of all known protein-protein interactions is associated with its biological processes of gene ontology (GO) derived from UniProtKB and Entrez Gene [30]–[32]. All ancestors of the GO tree are assigned in addition to achieve a complete and consistent annotation. In total, the human interaction network consists of 6390 different BP terms. 8939 (78%) nodes of the human interactome are at least associated with one biological process. A uniform functional sub-network is a connected fraction of the interactome, in which all the proteins share the same function, i.e., one unique GO term. The interactome can contain multiple disjoint functional sub-networks for the same annotation.

### Drug target profile

The recently published drug target profile of the kinase inhibitor bafetinib measured in the cell line K562 is used [33]. Rix et al took three quality criteria into account: (1) The drug target profile is devoid of proteins in the K562 core proteome. (2) No frequent hitters are included. (3) The proteins must be seen in replicates. In addition, splice variants and protein fragments are excluded. The 33 proteins are listed in Table S1.

### Perturbation of function

**Figure 2:**
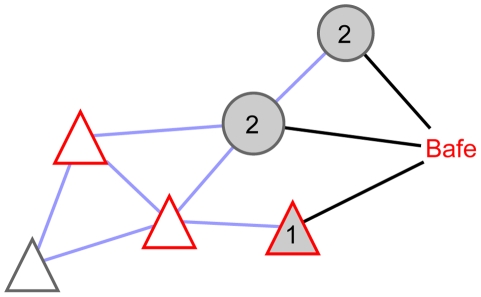
The perturbed functional network is a protein-protein interaction (blue edges) network. This network includes the uniform functional sub-network (triangular nodes) which shares one biological function and all drug targets (grey nodes) interacting with it. Bafetinib (Bafe) can impact nodes (red border) in the uniform functional sub-networks in two ways: Either the drug inhibits directly a node in the uniform functional sub-network (1) or it modulates the function through peripherally interacting drug targets (2).

Bafetinib can impact the uniform functional sub-networks in two ways via its targets (Figure 2):

1. The drug inhibits directly a node of the uniform functional sub-network.
2. The drug target interacts with the uniform functional sub-network at its periphery. To consider also complexes and cascades, drug targets which are linked via other drug targets to the functional sub-network are included.

**Figure 3:**
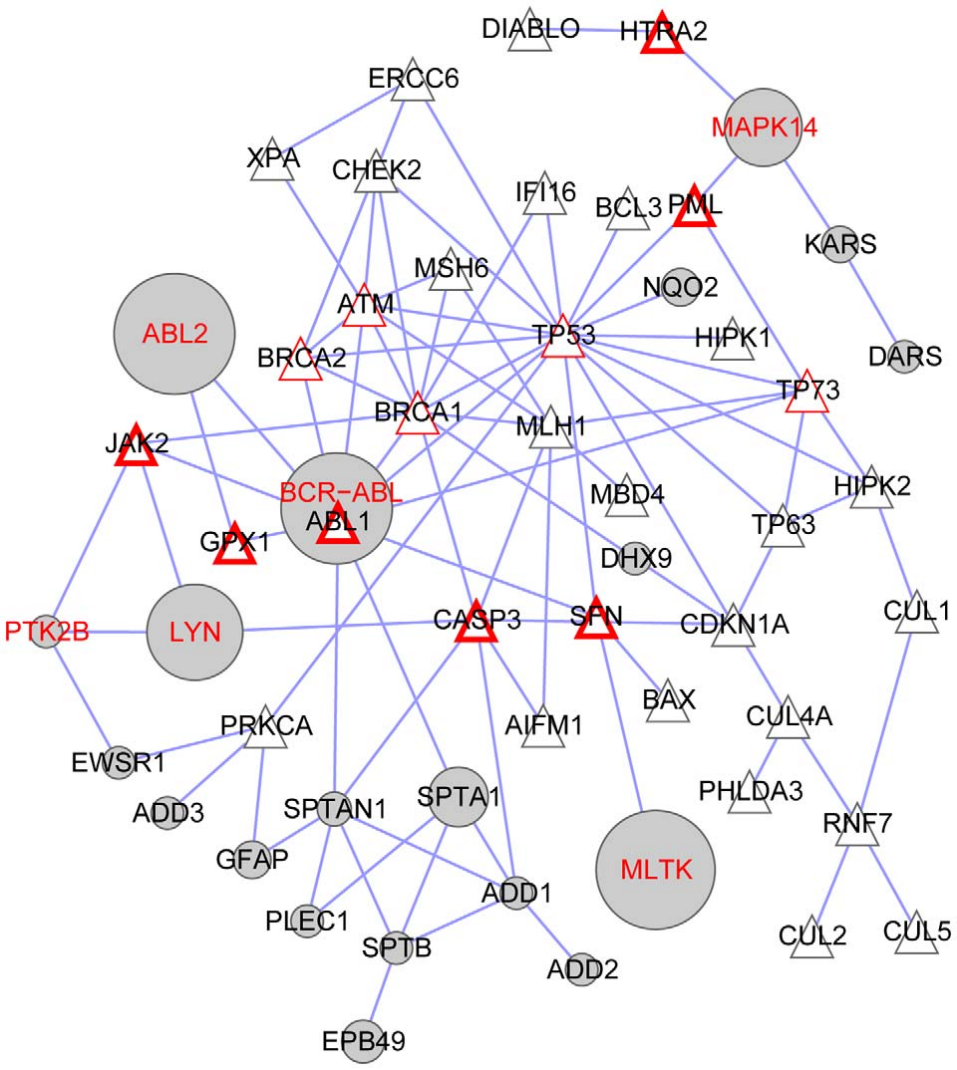
Perturbed functional sub-network based on induction of apoptosis by intracellular signals. In the protein-protein interaction (edges) network, the Bafetinib profile (grey nodes) perturbs the biological process (triangular nodes) which is pivotal in BCR-ABL dependent CML. The drug affinity (a_t_) is indicated by the node size. Kinases in the target profile have a red label. Proteins of the uniform functional sub-network interacting with inhibited kinases are shown with a red node border. K562 cells contain ABL1 and its fusion protein BCR-ABL which is not found by the algorithm in this sub-network. However, ABL1 pulldown is hidden by BCR-ABL and hence missed as target. Western plots proved ABL1 as a competed target [33] and hence influences are indicated with thin red borders.

It is difficult to predict which mode of perturbation has a higher impact. Directly inhibiting a pivotal sub-network member can completely disrupt a function. Nonetheless, biological signaling networks often have multiple alternative routes and protein isoforms to rescue the cell. Drug targets acting at the periphery can modify significantly the function through interaction or modulation of a modification, e.g., phosphorylation. By this mechanism, the inactivation of different branches and isoforms is possible. Furthermore, functional boundaries are often loosely defined and incompletely annotated. We thus treat both perturbation modes equally and therefore the perturbed functional sub-network (Figure 2) combines the uniform functional sub-networks with all interactions to the peripheral drug targets. This combination could result in joining otherwise disjoint uniform functional sub-networks via a drug target as linker (see MAPK14 in Figure 3). The direct targets are already members of the uniform functional sub-networks.

### Scoring the impact of bafetinib

We define a score *s*_net_ which predicts how strong functional sub-networks are perturbed by bafetinib. For this purpose different features of the perturbed functional sub-network are combined.

The first feature describes how frequent the annotation is present in the sub-network. Peripheral drug targets don't share the functional annotation (Figure 2), hence they dilute the functional annotation of the sub-networks. To ensure that the function is not underrepresented in the network, a first factor of the score is the ratio of the number *n*_annot,net_ of nodes which have a specific annotation to the total size *n*_net_ of the perturbed functional sub-network.

The second feature puts the drug impact in relation to the sub-network size. Generic biological functions result in very big sub-networks, in which the drug targets play overall no important role anymore. Furthermore, the drug should preferentially perturb a function at several different points. Hence, the proportion of the number *n*_drug,net_ of drug target nodes to the number *n*_net_ of all nodes in the perturbed functional sub-network resembles a good measure.

Lastly, the binding affinity *a_t_* of bafetinib to its targets or the potency of inhibition is important for effective perturbation. In theory, the affinities can be measured in biochemical assays which are not always available. However, we propose hereafter an *ad hoc* affinity measure derived from chemical proteomics data directly. The impact is summarized in the sum of drug affinities to its targets in the perturbed functional sub-network *T*_drug,net_ divided by the overall affinity of all possible drug targets *T*_durg_. Combining these factors results in a score for each disrupted functional network:
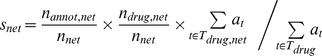
(1)The last two factors of equation (1) have an additional role and benefit. Mass spectrometry detection as used in chemical proteomics does not detect direct drug interactors only; it can also detect secondary interactors, i.e. proteins that bind to direct drug interactors. Without prior knowledge, it is difficult to distinguish between direct and indirect interactors but we believe that it is advantageous to use the complete target profile of bafetinib as it embeds the true drug targets into a specific context and increases the crosstalk with annotated nodes of the sub-networks (the second factor in equation (1) increases). The affinity factor ensures that also true and strong drug targets are part of the sub-network.

### Affinity score *a_t_* for chemical proteomics

The affinity of bafetinib to its targets is used to score the impact on sub-networks in equation (1). The higher the protein amount in mass spectrometry analysis, the higher the number of different detected peptides covering the protein sequence [34]. Hence, the peptide count *p*_t_ of each protein is a rough estimate of the amount of pulled-down protein. If soluble bafetinib is supplemented, the soluble drug blocks the binding pocket of its target yielding a reduced amount of pulled-down proteins that are specific drug binders (Figure 1) and thus, their peptide counts *p*_t,comp_ decrease. This observation is expressed in the first factor of the affinity score. Since in chemical proteomics the drug is always present at a large excess of constant concentration, it is only possible to distinguish the affinities of completely competed proteins by taking the protein amount into account. To down weigh this parameter influence, the logarithm is applied to *p_t_*. Thus, the affinity score can be expressed by the following equation:
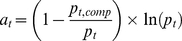
(2)Due to the reduced complexity of the competed pull-down, it can sporadically happen that *p*_t,comp_>*p_t_*. This case is seen as no competition and thus the affinity is set to 0.

### Randomization

An empirical p-value is calculated via randomization of the interactome. First, the interaction partners of each node are randomly selected. It is ensured that the degree of each node remains constant. Second, the annotation is randomly assigned to the nodes, while the total number of each term is preserved. The presented algorithm is applied to 500 random instances of the interactome. The empirical p-value is calculated from the fraction of randomized interactomes containing a sub-network with a score equal or better to the tested score divided by the total number of random instances. The highest score of all the random instances *s*_max, rand_ is 0.124.

### R-Package

The presented approach is programmed in the statistical environment R/Bioconductor and available at http://bioinformatics.cemm.oeaw.ac.at/drugDisruptNet [35], [36]. The provided R package depends on graph, RBGL, snow and GO.db [37]–[39]. Parallelization is done with snow to generate and score random instances [40]. Additionally, the above described data and the results are stored as R data objects.

### Visualization and comparison

Networks are visualized with Cytoscape [41]. For comparison, classical GO/KEGG/Biocarta enrichment analysis of sets are performed with DAVID [18].

## Results and Discussion

**Figure 4:**
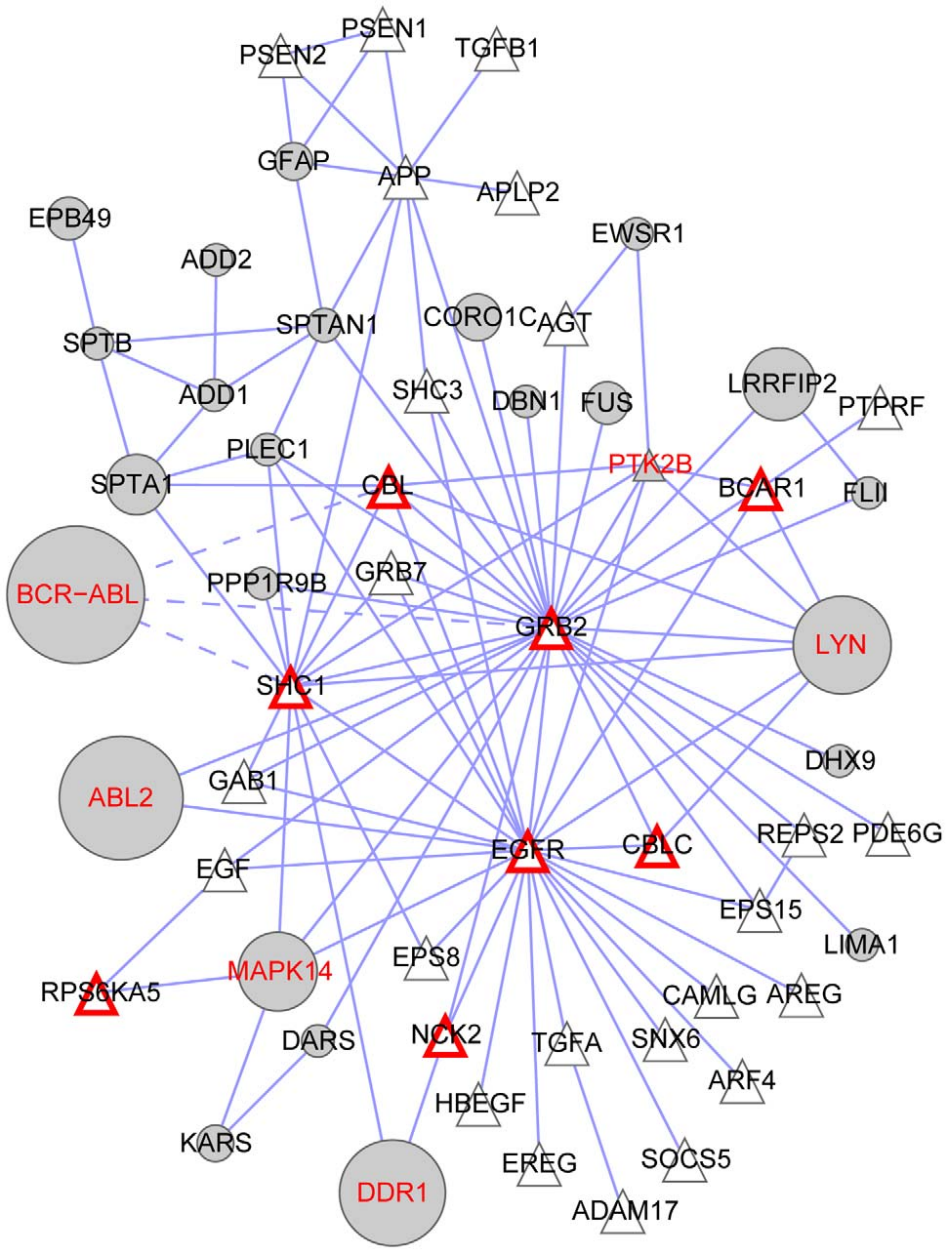
The bafetinib targets (grey nodes) perturb EGFR signaling which suggests a new application in lung cancer. The drug profile interferes with many nodes of the uniform function sub-network (triangular nodes). The drug affinity is indicated by the node size (a large node means high affinity). Kinases in the target profile have a red label. Proteins of the uniform functional sub-network interacting with inhibited kinases are shown with a red node border. EGFR expressing cells are not known to carry the fusion protein BCR-ABL which diminishes the influence of BCR-ABL on the network (dashed lines).

We present a novel strategy to analyze the mechanisms of action of bafetinib. The target profile is weighted with respect to its drug affinity and its impact on protein interaction networks is scored. Ten perturbed functional sub-networks are scored higher than any sub-network of the 500 randomized interactomes (*s*_max, rand_ = 0.124), see Table 1 and Figure 3, 4 and supplementary Figure S1, S2. The sub-networks do not necessarily contain all the components of a specific function since several disjoint functional sub-networks can be constructed. Bafetinib is designed to treat BCR-ABL dependent chronic myeloid leukemia (CML). Constitutively active BCR-ABL interferes strongly with apoptosis in malignant cells. We catch this process in our significantly perturbed sub-networks at rank 6 (Table 1). Furthermore, MAP kinase signaling can also be brought together with pathogenesis and treatment of CML. The top ranked perturbation of “Epidermal growth factor receptor (EGFR) signaling pathways” and “Insulin receptor signaling pathway” suggest potential novel domains of treatment for bafetinib and “heart development” indicates a putative side effect. The hit signaling pathways further play important roles in the general perturbed processes of aging, extracelluar structure organization and cell cycle. Finally, phosphorylation is an obvious process to be perturbed by a kinase inhibitor. We discuss pathogenesis, potential new domains of drug treatment and putative side effects of bafetinib in more details.

**Table 1:** Significantly perturbed functional sub-networks named by their basic function.

Perturbed functional sub-networks	Nodes	Targets (competed)	Score *s*_net_
Epidermal growth factor receptor signaling pathway	57	25 (7)	0.213
Insulin receptor signaling pathway	68	26 (7)	0.198
Aging	96	29 (7)	0.185
Regulation of MAP kinase activity	94	22 (7)	0.162
Induction of apoptosis by intracellular signals	53	19 (5)	0.135
Extracellular structure organization and biogenesis	81	21 (6)	0.135
MAPKKK cascade	188	29 (8)	0.131
Heart development	104	21 (7)	0.127
Protein amino acid autophosphorylation	71	21 (5)	0.126
Cell cycle arrest	73	21 (5)	0.125

(Randomization: N = 500; p-value<0.002).

Inactivated apoptosis signaling plays a pivotal role in BCR-ABL dependent CML pathogenesis [42] and is well represented in the significant sub-networks. The perturbed functional sub-network of apoptosis (Figure 3) is disrupted by inhibition of ABL2, MLTK, LYN and MAPK14 (p38α). These kinases are not annotated themselves as “induction of apoptosis by intracellular signals” but act at the periphery of the uniform functional sub-network. K562 cells express ABL1, a central node of the network, and its fusion protein BCR-ABL. High amounts of BCR-ABL hide specific ABL1 detection with mass spectrometry. However, western blots proved ABL1 as a competed target of bafetinib in K562 [33]. Hence, the score of perturbation underestimates the impact of bafetinib on apoptosis in CML.

The impact of bafetinib on apoptosis in CML is manifested with 5 targeted kinases at the periphery (Figure 3). The method strongly prefers networks which are attacked by several high affinity drug targets. In theory, a single perturbation might be enough to significantly interfere with a biological function. However, biological signaling networks are often highly redundant thus requiring perturbation at several points in order to observe an effect [43]. Hence, promiscuous drugs like dasatinib are very successful in CML and other cancers and the multi-targeted networks are likely to be of high relevance in drug treatment.

Even if we know that the drug has an inhibitory effect on the target kinases, we cannot predict without additional knowledge whether missing phosphorylation has an enhancing or decreasing effect on the biological process. The constitutively active kinase BCR-ABL results in a strong anti-apoptotic phenotype. Inhibition counteracts this behavior [44]. Inhibition of LYN has a similar effect in this context [45]. Contrary to this, MAPK14 inhibition rescues cells from apoptosis [46]. Only through the complex interplay of different signals, the malignant cells die upon treatment as desired. Hence, visualization of the network together with its disturbers strongly aids in interpreting their influence. This is a great advantage compared to simple GO enrichment analysis which does not display the relationship of the proteins to each other.

The top ranked perturbed functional sub-network is based on the epidermal growth factor receptor (EGFR) signaling pathway (Figure 4). It is peripherally interacting with six kinases of the drug profile. Three additional kinases are directly interacting with EGFR but also interfering with 7 further proteins of the signaling cascade. Additionally, the crosstalk between the pulled down non-kinase members and the functional network is very high. In total 13 out of 33 EGFR signaling components (39%) are interacting with the drug profile.

EGFR is not expressed in hematopoietic cells (such as K562) but this sub-network strongly suggests that bafetinib has the potential to interfere with EGFR signaling for instance in lung cancer cells. Recently, it was shown through the combination of chemical proteomics, phosphoproteomics and functional genomics that dasatinib, a broad-spectrum kinase inhibitor, leads to apoptosis in lung cancer cells via inhibition of SRC, EGFR, FYN and, notably, LYN [47]. Therefore, it is possible that also bafetinib might have a pro-apoptotic effect on these cells as it is also a potent inhibitor of LYN. While expression of dasatinib-insensitive gatekeeper mutants of DDR1 (or ABL1) did not rescue the H292 lung cancer cell line from dasatinib action, the role of DDR1 might be quite different in primary lung cancer cells as several recent reports described this receptor tyrosine kinase to be one of the most highly expressed and phosphorylated kinases in primary lung tumor specimens [48], [49]. Thus, it is conceivable that bafetinib might exert pro-apoptotic effects on lung cancer cells, and it might do so through simultaneous inhibition of LYN and DDR1.

Second highest is the perturbation of insulin receptor signaling pathway. It was suggested that bafetinib, CGP76030 and nilotinib might overcome imatinib resistance in blast crisis patients which feature BCR-ABL gene amplification [50]. Phase 1 studies could not verify this yet [51]. However, we propose to treat only the subgroup of CML blast crisis patients which expresses IGF1R with bafetinib. The drug targets are strongly interacting with the insulin receptor signaling pathway which maintains survival of hematopoietic cells through IGF1R (supplementary Figure S1). The IGF1R expression frequency is strongly increased in blast crisis patients (73%). Inhibition of IGF1R was shown in imatinib-resistant CML to induce apoptosis [52]. IGF1R is not a known direct target of bafetinib but attacking several downstream components simultaneously might show a similar effect as a direct IGF1R inhibition.

A potential side effect of several tyrosine kinase inhibitors, like sunitinib and dasatinib, is an increased risk for cardiotoxicity [53]. Observed toxicity in rats can be a result of higher concentration than used in patients [54]. Nevertheless, perturbation of the “heart development” network (Figure S2) indicates some possible risks which should be closely monitored during clinical trials.

We validated the robustness of the algorithm by following the rank of the biological process upon leaving-one-out (supplementary Figure S3). The ranks of the first five sub-networks (Table 1) are generally stable upon loss of a node. High affinity targets are essential to the phenotype which results in increased sensitivity of highly ranked terms to high affinity targets. On the contrary, weaker binders, which are not competed away with free drug, have only a modest effect on the rank.

Furthermore, we investigated the effect of hubs on the sub-network ranks, which might exert an influence on the phenotype upon inhibition. It is not clear whether hubs are, in the context of our analysis, highly important or “general signal diluters”. Therefore, we weighted up and then down the affinity of the targets by log10 of their node degree. Multiply by this factor, i.e. increasing hubs importance, the top 6 sub-networks remain unchanged and “cell cycle arrest” even improved its rank by one. The others sub-networks were substituted by “response to insulin stimulus”, “response to peptide hormone stimulus” and “cellular response to hormone stimulus”, which are in line with insulin receptor signaling. Upon down-weighting by division, the top 4 sub-networks still remained unchanged. We conclude for robustness and reasonable independence of local topology. In other words, the function of the sub-network at hand seems to play a strong role in scoring, which is appropriate.

To show the general interest of our method we applied the algorithm to data we published recently analyzing lung cancer (HCC297) treatment with dasatinib [47], another kinase inhibitor. Interestingly, dasatinib is highly promiscuous and pulls down 176 proteins (33 kinases) compared to the 33 proteins of bafetinib. In addition, free compound competition data were not available in this case; we thus exploited IC50s of autophosphorylation, which were available instead (Table S2A of Ref. [47]). The log10 of the IC50s (in the nM range) were used to weight the effect of dasatinib on the kinases. Analysis results in 681 significantly hit sub-networks due to the huge kinase profile (P-value<0.002, Table S2). Even though more than 5% of the human kinases are targeted by dasatinib and, subsequently, many sub-networks are significantly impacted, the top disrupted sub-networks are insightful. For instance, the top 10 ranked biological processes are centered on cell cycle arrest, cell growth and apoptosis. In the highest ranked sub-networks, SRC, LYN and EGFR play a pivotal role, which is absolutely consistent with our experimental data where these three proteins were shown with dasatinib gate-keeper mutants to strongly contribute to cell viability of HCC297 [47]. These results show that the algorithm can provide informative data even in very challenging situations.

In comparison to our approach, classical GO enrichment analysis (p-value<0.01) of the 33 bafetinib drug targets result in 33 significant biological processes with high redundancy in the GO tree (supplementary Table S3). Basically, they represent 3 GO terms: cytoskeleton organization (especially actin filament), phosphorylation and regulation of stress-activated protein kinase signaling pathway. Except phosphorylation which is obvious in a target profile of a kinase inhibitor there is no overlap with the perturbed functional sub-networks. The GO term of cytoskeleton organization contains competed and non-competed members of the target profile. The combined attack power of few competed kinases is too low to see perturbation of the large uniform functional sub-network (387 members) which is based on cytoskeleton organization. Enrichment analysis with KEGG and Biocarta pathways (p-value<0.01) yielded no hit.

In contrast to GO enrichment analysis, the presented method does not as much rely on accurate annotations. Possible missing annotations of drug targets interacting at the periphery with a functional sub-network have only a minor effect on the score. However, we would like to point out that boundaries of pathways and biological processes are very diffuse. Crosstalk between different signaling cascades and metabolic pathways is essential for a living cell. Integrating protein interactions to peripheral drug targets provides a way out of this dilemma and can catch therefore more relevant processes than GO enrichment. Alternatively, augmenting the drug target profile with their direct interactors, results in a set of 831 proteins. GO enrichment analysis (p-value<0.01) of this set results in 676 biological processes (Table S4). Again the first hits are related to general phosphorylation which is obvious for a kinase inhibitor profile. At the ninth rank “regulation of programmed cell death” which is related to perturbed apoptosis is presented with 127 proteins of the augmented set. The highest scored perturbed sub-network of EGFR signaling is only found at position 368. Even though the disrupted processes are detected with the augmented GO analysis, their ranks are so bad that they would not be considered as relevant. Our approach thus picks the most relevant perturbed functional networks and allows for insights beyond traditional GO enrichment analysis.

Competition experiments in chemical proteomics provide an additional layer of security to the drug target profile. Secondary and unspecific binders are difficult to distinguish from true drug targets. They are often similar in the range of peptide counts and other properties. The competition with a soluble drug and our affinity score helps in identifying biological target proteins. Interestingly, unspecific binders influence the perturbation algorithm only marginally since the proteins are dispersed all-over the interactome and have no affinity to a specific uniform functional sub-network. Furthermore, their binding affinity score is 0. On the contrary, secondary binders of true drug targets increase the crosstalk to the functional sub-network which is attacked by the true target. Hence they can be used advantageously embedding the true targets in a specific context.

In conclusion, we identified successfully known mechanisms in CML as well as potential new applications and possible side-effects. We believe that the proposed computational approach can shed light in mechanisms of other drugs including highly promiscuous compounds and when soluble compound competition data are lacking. Hence, we provide an R package at http://bioinformatics.cemm.oeaw.ac.at/drugDisruptNet.

## Supporting Information

Figure S1 The bafetinib targets (grey nodes) disrupt the insulin receptor signaling pathway. The drug profile interferes with many nodes of the uniform function sub-network (triangular nodes). The drug affinity is indicated by the node size (large node equals high affinity). Kinases in the target profile have a red label. Proteins of the uniform functional sub-network interacting with inhibited kinases are shown with a red node border.
(0.47 MB TIF)

Figure S2 The bafetinib targets (grey nodes) disrupt the heart development suggesting putative risk factors. The drug profile interferes with many nodes of the uniform function sub-network (triangular nodes). The drug affinity is indicated by the node size (large node equals high affinity). Kinases in the target profile have a red label. Proteins of the uniform functional sub-network interacting with inhibited kinases are shown with a red node border.
(0.49 MB TIF)

Figure S3 Leave-one-out analysis. The ranks of the first five subnetworks (Table 1) are generally stable upon loss of a node. High affinity targets (left) are essential to the phenotype which results in increased sensitivity of highly ranked terms to high affinity targets. On the contrary, weaker binders (right) have only a modest effect on the rank.
(0.11 MB PDF)

Table S1 Bafetinib (INNO-406) drug target profile.
(0.06 MB DOC)

Table S2 Significantly hit sub-network by the highly promiscuous drug dasatinib.
(0.12 MB XLS)

Table S3 Classical gene ontology (GO) enrichment analysis (p-value<0.01). The secondary and unspecific binders have a large influence on GO enrichment analysis.
(0.04 MB DOC)

Table S4 Classical gene ontology (GO) enrichment analysis on all direct interactors of the drug profile (p-value<0.01).
(0.40 MB XLS)
