# Supplementary figures and images for: A Computational Approach to Analyze the Mechanism of Action of the Kinase Inhibitor Bafetinib
Source: PLoS Comput Biol. 2010 Nov 18;6(11):e1001001. doi: 10.1371/journal.pcbi.1001001 (PMC2987840; doi:10.1371/journal.pcbi.1001001)

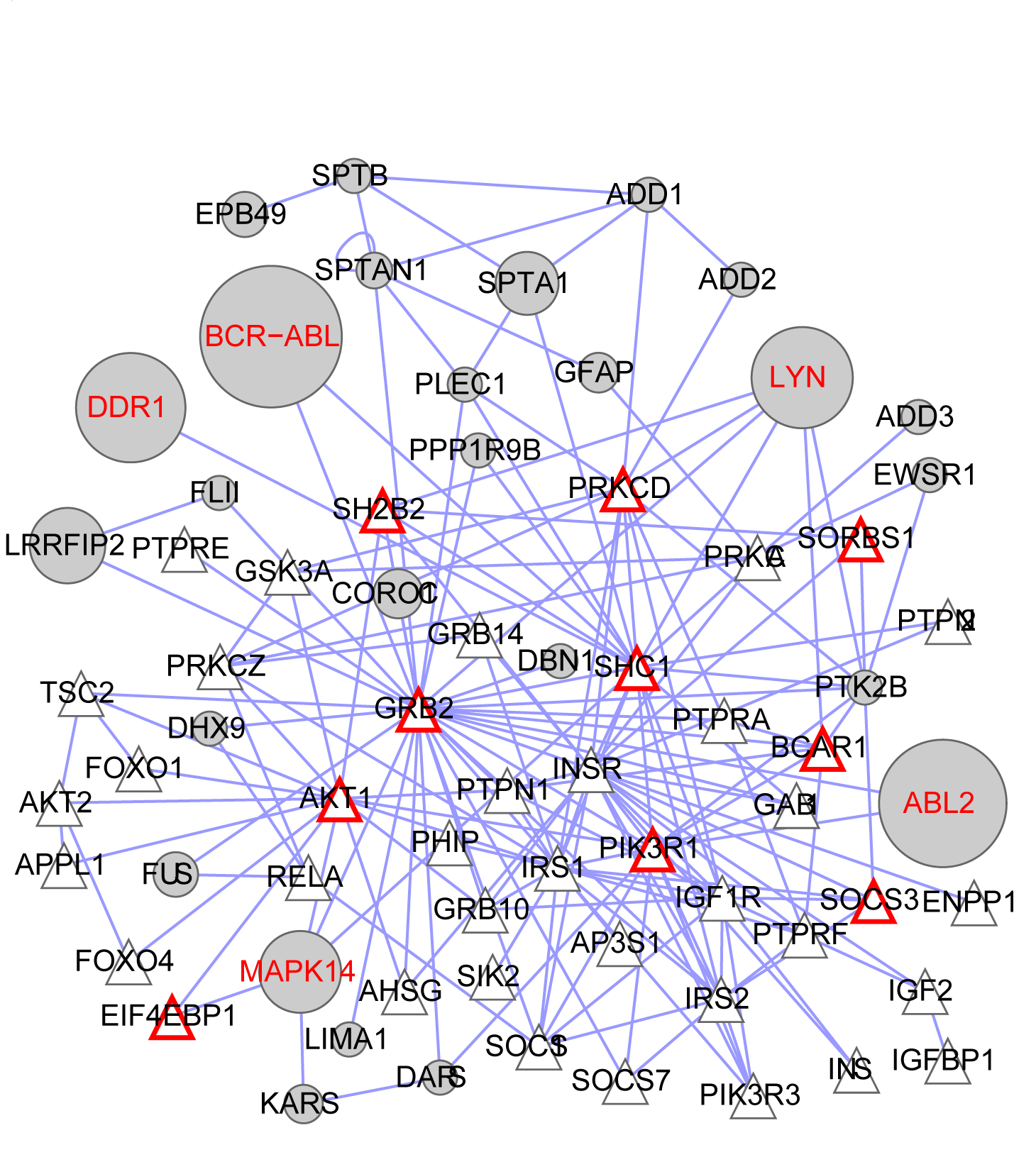

Supplement: Figure S1 — The bafetinib targets (grey nodes) disrupt the insulin receptor signaling pathway. The drug profile interferes with many nodes of the uniform function sub-network (triangular nodes). The drug affinity is indicated by the node size (large node equals high affinity). Kinases in the target profile have a red label. Proteins of the uniform functional sub-network interacting with inhibited kinases are shown with a red node border. (0.47 MB TIF) [file pcbi.1001001.s001.tif]

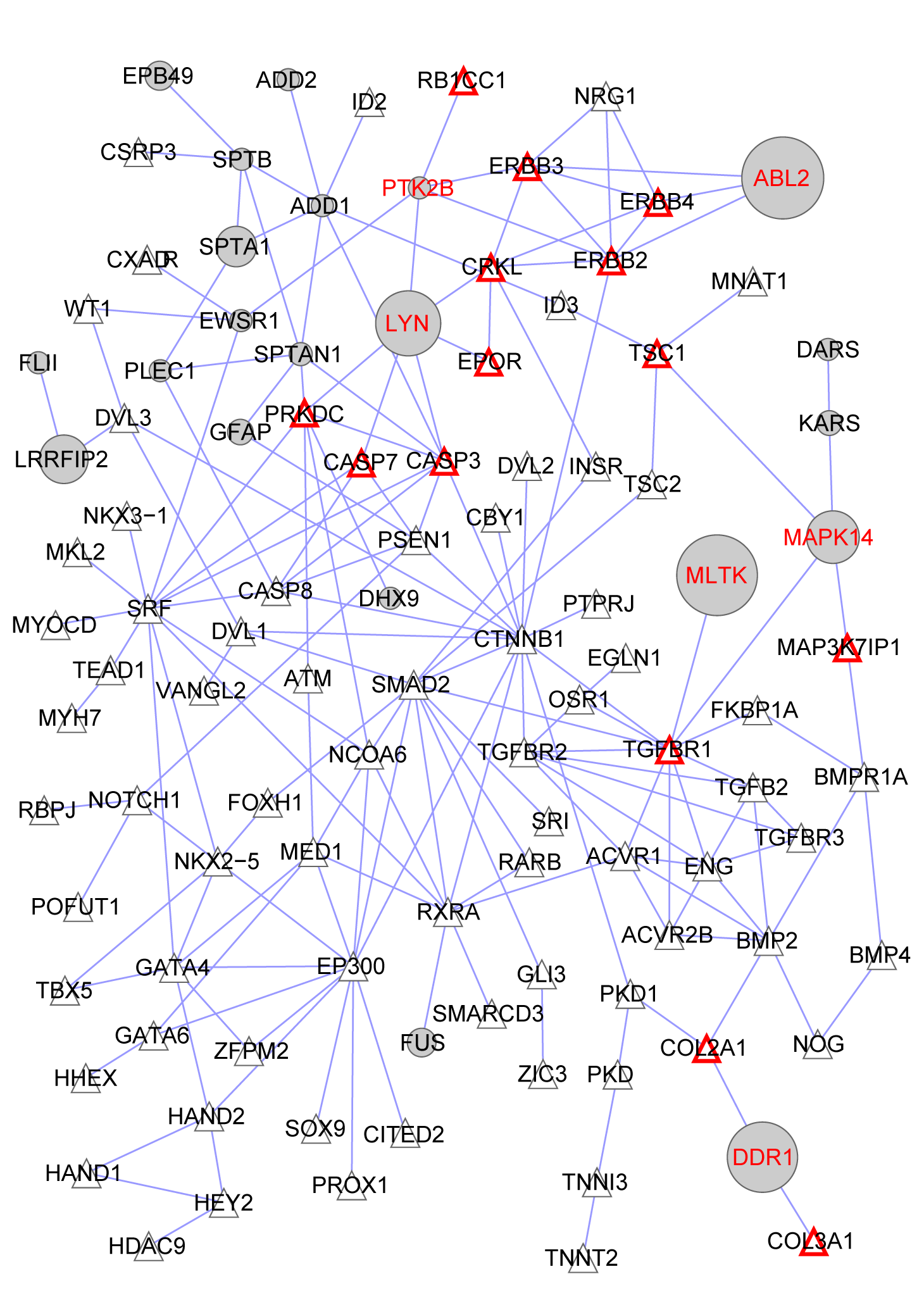

Supplement: Figure S2 — The bafetinib targets (grey nodes) disrupt the heart development suggesting putative risk factors. The drug profile interferes with many nodes of the uniform function sub-network (triangular nodes). The drug affinity is indicated by the node size (large node equals high affinity). Kinases in the target profile have a red label. Proteins of the uniform functional sub-network interacting with inhibited kinases are shown with a red node border. (0.49 MB TIF) [file pcbi.1001001.s002.tif]

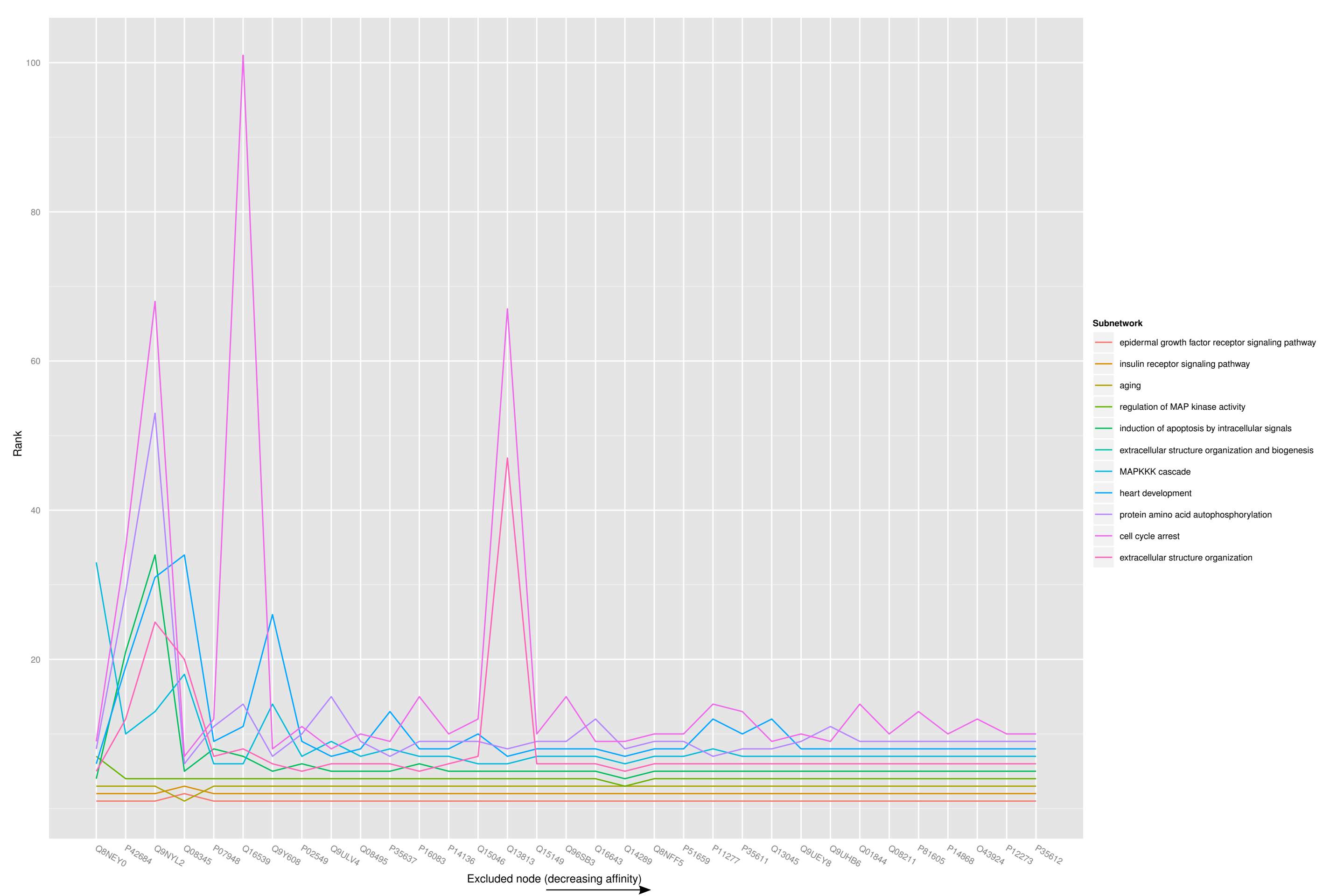

Supplement: Figure S3 — Leave-one-out analysis. The ranks of the first five subnetworks (Table 1) are generally stable upon loss of a node. High affinity targets (left) are essential to the phenotype which results in increased sensitivity of highly ranked terms to high affinity targets. On the contrary, weaker binders (right) have only a modest effect on the rank. (0.11 MB PDF) [file pcbi.1001001.s003.pdf]
