# Supplementary material for: A Computational Approach to Analyze the Mechanism of Action of the Kinase Inhibitor Bafetinib
Source: PLoS Comput Biol. 2010 Nov 18;6(11):e1001001. doi: 10.1371/journal.pcbi.1001001 (PMC2987840; doi:10.1371/journal.pcbi.1001001)
Supplement: Table S1 — Bafetinib (INNO-406) drug target profile. (0.06 MB DOC) [file pcbi.1001001.s004.doc]

**Table S1.** Bafetinib (INNO-406) drug target profile [1]; the drug targets are devoid of proteins in the K562 core proteome and frequent hitter list. Furthermore, they must be seen in replicates. Isoforms and protein fragments are excluded. Peptide counts are averaged over replicates. Fields: UniProtKB AC, peptide count of drug targets (*p_t_*); peptide count of drug targets in the competition experiment (*p_t,comp_*)

| UniProtKB AC | Gene Symbol | *p_t_* | *p_t,comp_* |
| --- | --- | --- | --- |
| Q8NEY0 | BCR-ABL | 37 | 0 |
| P42684 | ABL2 | 23 | 0 |
| Q9NYL2 | MLTK | 21.5 | 0 |
| Q08345 | DDR1 | 12.5 | 0 |
| P07948 | LYN | 9.5 | 0 |
| Q9Y608 | LRRFIP2 | 4 | 0 |
| Q16539 | MAPK14 | 19.5 | 9 |
| P02549 | SPTA1 | 5 | 2 |
| Q13813 | SPTAN1 | 82 | 81 |
| Q15149 | PLEC1 | 29.5 | 97 |
| Q96SB3 | PPP1R9B | 25 | 26 |
| Q16643 | DBN1 | 13.5 | 15 |
| Q9ULV4 | CORO1C | 17 | 14 |
| P16083 | NQO2 | 14 | 13 |
| Q14289 | PTK2B | 12 | 13 |
| Q8NFF5 | FLAD1 | 11 | 11 |
| P51659 | HSD17B4 | 8 | 13 |
| Q15046 | KARS | 6.5 | 6 |
| Q9UHB6 | LIMA1 | 4.5 | 9 |
| P11277 | SPTB | 6 | 7 |
| P35611 | ADD1 | 6 | 9 |
| Q13045 | FLII | 5.5 | 7 |
| Q9UEY8 | ADD3 | 5 | 5 |
| P35637 | FUS | 4 | 3 |
| Q01844 | EWSR1 | 4 | 5 |
| P81605 | DCD | 3.5 | 4 |
| P14868 | DARS | 3 | 4 |
| Q08211 | DHX9 | 4 | 10 |
| Q08495 | EPB49 | 3 | 2 |
| P14136 | GFAP | 2.5 | 2 |
| O43924 | PDE6D | 2.5 | 3 |
| P12273 | PIP | 2 | 3 |
| P35612 | ADD2 | 2 | 5 |

1. Rix U, Remsing Rix LL, Terker AS, Fernbach NV, Hantschel O, et al. (2010) A comprehensive target selectivity survey of the BCR-ABL kinase inhibitor INNO-406 by kinase profiling and chemical proteomics in chronic myeloid leukemia cells. Leukemia 24: 44-50.
