# Supplementary material for: A Computational Approach to Analyze the Mechanism of Action of the Kinase Inhibitor Bafetinib
Source: PLoS Comput Biol. 2010 Nov 18;6(11):e1001001. doi: 10.1371/journal.pcbi.1001001 (PMC2987840; doi:10.1371/journal.pcbi.1001001)
Supplement: Table S3 — Classical gene ontology (GO) enrichment analysis (p-value<0.01). The secondary and unspecific binders have a large influence on GO enrichment analysis. (0.04 MB DOC) [file pcbi.1001001.s006.doc]

**Table S3.** Classical gene ontology (GO) enrichment analysis performed with DAVID [1] (p-value < 0.01); The secondary and unspecific binders have a large influence on GO enrichment analysis.

| GO Term (biological process [GOTERM_BP_FAT]) | P-Value |
| --- | --- |
| Actin filament organization | 8.83E-13 |
| Negative regulation of actin filament depolymerization | 4.57E-12 |
| Regulation of actin filament depolymerization | 1.00E-11 |
| Negative regulation of protein complex disassembly | 1.49E-10 |
| Actin filament bundle formation | 4.26E-10 |
| Actin filament capping | 5.51E-10 |
| Regulation of protein complex disassembly | 5.90E-10 |
| Negative regulation of cytoskeleton organization | 9.45E-10 |
| Regulation of actin polymerization or depolymerization | 1.80E-09 |
| Negative regulation of actin filament polymerization | 2.04E-09 |
| Regulation of actin filament length | 2.19E-09 |
| Negative regulation of protein polymerization | 2.46E-09 |
| Negative regulation of protein complex assembly | 5.72E-09 |
| Actin cytoskeleton organization | 9.05E-09 |
| Negative regulation of organelle organization | 1.10E-08 |
| Actin filament-based process | 1.50E-08 |
| Regulation of actin cytoskeleton organization | 1.81E-08 |
| Regulation of actin filament-based process | 2.21E-08 |
| Regulation of cellular component size | 3.73E-08 |
| Regulation of actin filament polymerization | 6.33E-08 |
| Regulation of protein polymerization | 2.05E-07 |
| Regulation of cytoskeleton organization | 2.30E-07 |
| Negative regulation of cellular component organization | 2.97E-07 |
| Regulation of protein complex assembly | 8.38E-07 |
| Cytoskeleton organization | 1.41E-06 |
| Regulation of organelle organization | 3.55E-06 |
| Regulation of cellular component biogenesis | 7.96E-06 |
| Peptidyl-tyrosine phosphorylation | 1.02E-04 |
| Peptidyl-tyrosine modification | 1.16E-04 |
| Regulation of protein amino acid phosphorylation | 0.004798 |
| Regulation of stress-activated protein kinase signaling pathway | 0.008288 |
| Protein amino acid phosphorylation | 0.009388 |
| Barbed-end actin filament capping | 0.009941 |

1. Huang da W, Sherman BT, Lempicki RA (2009) Systematic and integrative analysis of large gene lists using DAVID bioinformatics resources. Nat Protoc 4: 44-57.
